# The Predictive Value of Serum Uric Acid on Acute Kidney Injury following Traumatic Brain Injury

**DOI:** 10.1155/2020/2874369

**Published:** 2020-08-31

**Authors:** Ruo Ran Wang, Min He, Xiao Feng Ou, Xiao Qi Xie, Yan Kang

**Affiliations:** Department of Critical Care Medicine, West China Hospital, Sichuan University, Chengdu, 610041 Sichuan Province, China

## Abstract

**Backgrounds:**

Acute kidney injury (AKI) is a prevalent nonneurological complication in patients with traumatic brain injury (TBI). We designed this study to explore the association between serum uric acid (SUA) level and the occurrence of AKI following TBI.

**Methods:**

This is a retrospective single-center study. A total of 479 patients admitted with TBI were included in this study. We utilized SUA and other risk factors for AKI to construct a predictive model by performing multivariate logistic regression. 374 patients and 105 patients were, respectively, divided into a training set and validation set. The predictive value of the single SUA and constructed model was evaluated by drawing a receiver operating characteristic (ROC) curve. AKI was diagnosed according to the KIDGO criteria.

**Results:**

79 (21.12%) patients were diagnosed with AKI in the training cohort. The patients in the AKI group are older than those in the non-AKI group (*p* = 0.01). And the Glasgow Coma Scale (GCS) of the AKI group was lower than that of the non-AKI group (*p* < 0.001). In a multivariate logistic regression analysis, we found that heart rate (*p* = 0.041), shock (*p* = 0.018), serum creatinine (*p* < 0.001), and serum uric acid (SUA) (*p* < 0.001) were significant risk factors for AKI. Bivariate correlation analyses showed that serum creatinine was moderately positively correlated with SUA (*r* = 0.523, *p* < 0.001). Finally, the area under the receiver operating characteristic curve (AUC) of SUA for predicting AKI in the training set and validation set was 0.850 (0.805-0.895) and 0.869 (0.801-0.938), respectively.

**Conclusions:**

SUA is an effective risk factor for AKI following TBI. Combining SUA with serum creatinine could more accurately identify TBI patients with high risk of developing AKI.

## 1. Introduction

It has been estimated that 10 million people would suffer a traumatic brain injury (TBI) event every year all over the world [1]. More than 1.4 million people may undergo TBI annually in the USA, and 50,000 of them will die due to this kind of severe injury [2]. In addition to the neurological damage of TBI, nonneurological complications could also contribute to the mortality and unfavored outcome [3]. Acute kidney injury, a form of nonneurological complications, has been paid much attention in a neurointensive care unit (NICU). Both pathophysiological effects of secondary brain injury and side effects of therapy could aggravate the development of AKI after TBI [4]. Previous studies have shown that the incidence of AKI following TBI ranged from 3.9% to 24% [5–8]. And the association between occurrence of AKI and increased mortality and prolonged length of hospital stay (LOS) has been verified [5, 9, 10]. To avoid the adverse effect of AKI on the outcome of TBI patients, predicting the development of AKI in an early stage is necessary for neurosurgeons and intensivists to reduce the use of drugs with high nephrotoxicity and convert patients to drugs which have less adverse effects on renal function. For reducing intracranial pressure, more glycerol fructose and hypertonic saline should be used rather than mannitol with relatively serious side effects on the kidney.

Uric acid, an end product of purine metabolism, has been confirmed to be neuroprotective because of its antioxidant property. Previous studies have shown that elevated serum uric acid (SUA) level was associated with a favorable outcome in patients with ischemic stroke, intracerebral hemorrhage, or TBI [11, 12]. However, the correlation between AKI, poor progression of chronic kidney disease (CKD), and hyperuricemia has been verified. Uric acid could impair renal tubular endothelial function and cause subtle renal damage by intratubular crystal precipitation as well as activation of oxidative stress and renal inflammation [13, 14]. There are several studies examining the association between SUA and the occurrence of AKI in various patients including those undergoing radical cystectomy and those undergoing cardiac surgery [15–17]. However, the predictive value of SUA on AKI following TBI has not been examined. We performed this study to determine whether SUA was valuable in predicting AKI in patients with TBI and explored the correlation between SUA and other factors.

## 2. Materials and Methods

### 2.1. Patients

This is a single-center cohort study. We included patients diagnosed with TBI and hospitalized in West China Hospital within 24 hours after injuries between January 2015 and October 2019. The diagnosis of TBI was established by analyzing medical history and clinical manifestation and was confirmed by reviewing images including computed tomography (CT) and magnetic resonance imaging (MRI). Neurosurgeons and intensivists provided patients with reasonable treatments according to the current guidelines. Patients simultaneously diagnosed with other central nervous system diseases, hematological system diseases, cardiovascular diseases, tumor diseases, metabolic diseases, chronic hepatorenal diseases, and pregnancy were excluded. Patients who lack laboratory and clinical data on the first day and those lost to follow-up were also excluded from this study. Finally, 479 patients were included in this retrospective observational study. To explore and verify the predictive value of risk factors and predictive models, 374 patients admitted between January 2015 and September 2018 were selected as a training cohort and 105 patients admitted between October 2018 and October 2019 were selected as a validation cohort. The study was approved by the ethics committee of West China Hospital. All patients provided written informed consent.

### 2.2. Data Collection

Records of prehospital time were collected by emergency workers. Vital signs and the Glasgow Coma Scale (GCS) in admission were collected through inquiring the electronic medical record (EMR) system. A shock state was evaluated and recorded by nurses on the first day of admission. SUA level and other laboratory indexes were obtained by analyzing blood specimens on the first day of admission. AKI was evaluated after 48 hours after admission according to the KIDGO criteria [18].

### 2.3. Statistical Analysis

Normally distributed data was presented as mean ± standard deviation while nonnormally distributed data was presented as median (interquartile range). The Kolmogorov-Smirnov test was used to confirm the normality of the included variables. Categorical data was presented as numbers (percentage). We used independent Student's *t*-test to compare the difference between two groups of normally distributed variables. And the Mann-Whitney *U* test was used to compare the difference between two groups of nonnormally distributed variables. The difference of categorical variables was analyzed by using the *χ*^2^ test. Multivariate logistic regression analysis was utilized to analyze the association between various factors and the occurrence of AKI. The odds ratio (OR) and 95% confidence intervals (CI) of each risk factor were also calculated. We performed Spearman's method to analyze the correlation of serum uric acid level and other laboratory variables. To testify the value of different models for predicting AKI, we have drawn the receiver operating characteristic (ROC) curve and calculated the area under the curve (AUC), sensitivity, and specificity. Finally, the *Z* test was utilized to test the difference of AUC.

A *p* value < 0.05 was considered to be of statistical significance. SPSS 22.0 Windows software (SPSS, Inc., Chicago, IL) was used for all statistical analyses.

## 3. Results

### 3.1. Baseline Characteristics of AKI Patients and Non-AKI Patients

As shown in Table 1, among 374 patients diagnosed with TBI in the training cohort, 79 (21.12%) patients developed AKI. The age of the AKI group was 47 (32-60) while that of the non-AKI group was 41 (23-54) (*p* = 0.01). The female percentage did not differ between the non-AKI group and the AKI group (*p* = 0.433). The prehospital time of the AKI group was shorter than that of the non-AKI group (*p* = 0.028). Comparing vital signs in admission, we found that the heart rate of the AKI group was higher (*p* = 0.015) and the respiratory rate of the AKI group was lower (*p* = 0.022) than those of the non-AKI group. The GCS score of all patients was 6 (5-9), indicating that the included patients were mostly diagnosed with moderate and severe TBI. The AKI group had significantly lower GCS than the non-AKI group (*p* < 0.001). In addition, patients in the AKI group were more likely to develop a shock state than patients in the non-AKI group (*p* < 0.001). The results of the laboratory tests showed that only white blood cell (WBC) count was not significantly different between the two groups. The AKI group has higher level of glucose (*p* < 0.001), chlorine (*p* < 0.001), total bilirubin (*p* = 0.001), serum urea (*p* < 0.001), serum creatinine (*p* < 0.001), and SUA (*p* < 0.001). Instead, the non-AKI group has higher level of platelet (*p* < 0.001), hemoglobin (*p* < 0.001), albumin (*p* < 0.001), and cholesterol (*p* < 0.001). In consideration of several drugs for reducing intracranial pressure (ICP), only the utilization rate of hypertonic saline was different between these two groups (*p* = 0.004). Patients in the AKI group were less likely to receive hypertonic saline. Finally, patients who developed AKI could suffer increased in-hospital mortality (*p* < 0.001) and unfavorable 90-day GOS (*p* < 0.001). The median length of ICU stay in overall patients was 2 days. The length of ICU stay did not significantly differ between the non-AKI group and the AKI group (*p* = 0.384). The median length of hospital stay was 11 days. Compared with the non-AKI group, the AKI group had shorter length of hospital stay (*p* = 0.001).

### 3.2. Multivariate Logistic Regression Analysis of Risk Factors for AKI

Statistically significant variables in baseline comparison were selected for subsequent multivariate logistic regression analysis. As shown in Table 2, four factors including heart rate (OR 0.984, 95% CI [0.969-0.999]), shock (OR 2.905, 95% CI [1.201-7.028]), serum creatinine (OR 1.033% CI [1.017-1.048]), and SUA (OR 1.008, 95% CI [1.004-1.011]) were statistically significant.

### 3.3. Bivariate Correlation Analyses of Uric Acid and Various Clinical and Laboratory Parameters

Spearman's method was performed to analyze the correlation between other parameters and SUA. We found that heart rate (*r* = 0.163, *p* = 0.002), shock (*r* = 0.204, *p* < 0.001), WBC (*r* = 0.237, *p* < 0.001), glucose (*r* = 0.285, *p* < 0.001), chlorine (*r* = 0.286, *p* < 0.001), and serum urea (*r* = 0.291, *p* < 0.001) were weakly positively correlated with SUA (Table 3). And GCS (*r* = −0.204, *p* < 0.001), platelet (*r* = −0.264, *p* < 0.001), albumin (*r* = −0.126, *p* = 0.015), and cholesterol (*r* = −0.184, *p* < 0.001) were weakly negatively correlated with SUA. Furthermore, serum creatinine was moderately positively associated with SUA (*r* = 0.523, *p* < 0.001).

### 3.4. Comparison of the Predictive Value of Different Factors and Models to Predict AKI in the Training Cohort and Validation Cohort

**Figure 1:**
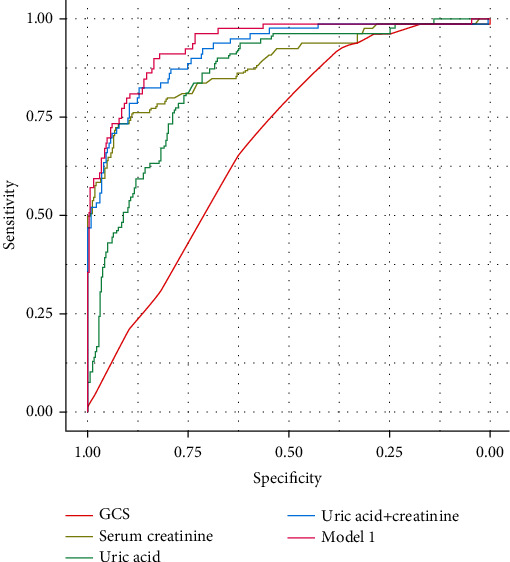
ROC curves of risk factors and constructed model 1 in the training cohort.

**Figure 2:**
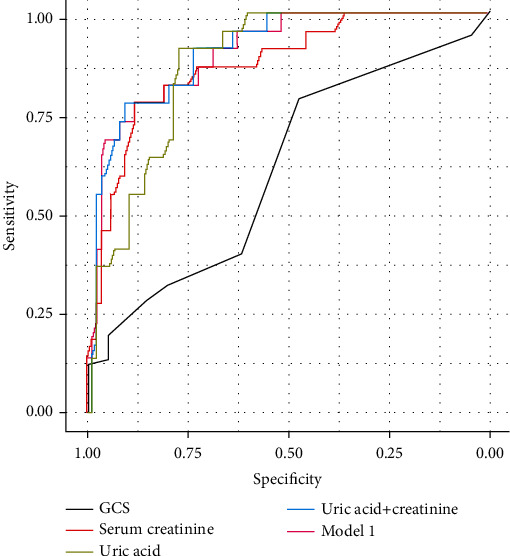
ROC curves of risk factors and constructed model 1 in the validation cohort.

In the training cohort, the AUC of SUA and serum creatinine was 0.850 and 0.881, respectively (Table 4) (Figure 1). There was no difference in AUC between SUA and serum creatinine (*Z* = 0.9126, *p* > 0.05). The AUC of combining SUA with serum creatinine was 0.917, which was higher than that of SUA alone (*Z* = 2.246, *p* < 0.05). Then, we use heart rate, shock, serum creatinine, and SUA which were statistically significant in multivariate logistic analysis, to construct predictive model 1. The AUC of model 1 was 0.935, which was not significantly higher than the AUC of combining SUA with serum creatinine (*Z* = 0.7247, *p* > 0.05). Finally, the AUC of GCS was 0.686, which was significantly lower than that of SUA (*Z* = 4.338, *p* < 0.05). In the validation cohort, the AUC of SUA and serum creatinine was 0.869 and 0.868, respectively (Table 4) (Figure 2). And the AUC of combining uric acid with creatinine was 0.905, which was higher than 0.900 of constructed model 1 and 0.869 of uric acid though without statistical significance (*Z* = 0.0996, *p* > 0.05; *Z* = 0.7273, *p* > 0.05). The AUC of GCS for predicting AKI was 0.610 in the validation cohort.

## 4. Discussion

The aim of this study was to confirm the predictive value of SUA in AKI following TBI. By performing multivariate logistic regression analysis, we found that heart rate in admission, shock, serum creatinine, and SUA were risk factors for the development of AKI. ROC curves showed that SUA had a comparable predictive value with serum creatinine alone. In our study, the percentage of patients developing AKI was 21.12% according to the KIDGO criteria. Previous studies reported that the incidence of AKI following TBI ranged from 0.45% to 35% [3, 19–21]. This variability might originate from different diagnostic criteria for AKI, severity of the included patients, and different medical level. We found that patients who developed AKI had higher age and lower GCS, which was consistent with the results of previous studies [6, 7]. Baseline comparison indicated that the occurrence rate of shock was higher in the AKI group. This was coincident with the opinion that developing AKI was usually aggravated by reduced renal perfusion pressure secondary to shock or hypovolemia [22]. In addition, the fact that the heart rate of the AKI group was higher than that of the non-AKI group could be explained by the complementary response to hypovolemia or the surge of catecholamine after brain injury. Most of significant laboratory parameters in baseline comparison showed no statistical significance in multivariate logistic regression analysis. Only serum creatinine and SUA were identified as risk factors for AKI. Moreover, bivariate correlation analysis showed that SUA was moderately correlated with serum creatinine. Therefore, we, respectively, draw the ROC curve of SUA and serum creatinine and found that there was no statistical difference in their AUC value. The single serum creatinine level was previously considered an essential element for diagnosing AKI. However, a single assessment of serum creatinine as reflection of renal filtration function is applicable for patients with a steady state but not for those critically ill patients. Influenced by fluid dilution and blood loss which commonly occur in critically ill patients, the single serum creatinine could not sensitively and promptly reflect the acute fluctuation of estimated glomerular filtration rate (eGFR). Recently, an increasing number of studies suggested that the change in renal function from baseline was better in assessing renal injury than single creatinine cutoffs [6, 23]. In our study, we found that combining SUA and serum creatinine could improve the accuracy of identifying patients with high risk of developing AKI after TBI. Considering drugs for reducing ICP, our study showed that the utilization rate of hypertonic saline was higher in the non-AKI group. Suitable use of hypertonic saline was beneficial for maintaining hemodynamic stability and renal perfusion pressure, which in turn decrease the probabilities of developing AKI. The percentage of patients using furosemide was higher in the AKI group, though without significance. This fact emphasized that unreasonable use of furosemide could lead to AKI due to hypovolemia. It has been demonstrated that accumulative doses of furosemide and mannitol were independent risk factors for AKI after cerebral trauma [24]. Nevertheless, our results did not show that the use of mannitol was associated with increased probabilities of AKI. This may be explained by the defect that chronological sequence of using mannitol and occurrence of AKI was not recorded. Finally, the fact that in-hospital mortality was obviously higher in the AKI group was consistent with previous studies. One cause of increased mortality in the AKI group was that renal dysfunction could aggravate cerebral edema [25]. In addition, patients who developed AKI had lower GCS which usually indicated higher severity of TBI. The 90-day GOS of the AKI group was undoubtedly lower than that of the non-AKI group. A finding that contradicted with previous studies was that AKI would shorten the length of hospital stay. A reasonable explanation was that the mortality of the AKI group in our study was too high to significantly shorten survival time.

The mechanism of systemic complications after TBI is diversified including direct effects of brain injury as well as side effects of therapy which could initiate or exacerbate systemic complications [26]. Massive release of catecholamine and neuroinflammation caused by brain injury may lead to systemic complications including AKI [4]. Furthermore, various therapy strategies targeted on maintaining and improving cerebral physiological parameters may concomitantly impair renal function.

In humans, uric acid is an end product of catabolism of purine nucleotides arising from endogenous and exogenous sources [27]. TBI triggers catabolic processes of a series of substances including adenosine triphosphate (ATP), a major part of endogenous purine nucleotides. Studies have illustrated that accumulating uric acid impaired renal function not only by intratubular crystal precipitation but also through inducing oxidative stress and renal inflammation [14]. Uric acid could activate the renin-angiotensin system, reduce nitric oxide (NO) release, and inhibit NO synthase 1. As a result, renal ischemia and hypertension secondary to renal vasoconstriction may accelerate the development of AKI [28]. Furthermore, through inducing the expression of C-reactive protein (CRP) and monocyte chemoattractant protein-1 (MCP-1) [29, 30] and stimulating the proliferation of vascular smooth muscle cells, uric acid plays a proinflammatory role in impairing autoregulation of renal blood flow and reducing the GFR [31, 32].

The predictive and therapeutic role of uric acid has been widely studied in brain injury patients including ischemic stroke, intracerebral hemorrhage, and TBI [12, 33, 34]. One study found that low SUA level within four hours after initial injury was associated with increased mortality in TBI patients [33]. Another study made a conclusion that low SUA level which indicated more consumption by injured brain tissue was beneficial for a favorable outcome in TBI patients [11]. And exogenous supplement of uric acid could improve sensorimotor functional recovery, spatial learning, and memory in CCI mice. However, the time range of SUA assessment in the latter study was extended to three days after initial injury. We speculated that the different time points of SUA assessment lead to the opposite conclusion in these two studies. Early low SUA level indicated no enough production of neuroprotective uric acid and therefore led to a poor outcome in TBI patients. But subsequent non-early-stage low SUA could indicate more consumption of uric acid which was associated with a favorable outcome in TBI patients. However, these two studies did not evaluate the effect of endogenous SUA level and exogenous uric acid administration on AKI following TBI. Previous studies have verified the association between SUA and the occurrence of AKI in various patients including those undergoing radical cystectomy and those undergoing cardiac surgery [15–17]. Our study demonstrated that high SUA was a potent risk factor for AKI following TBI. In addition, it is generally established that AKI is an independent risk factor for mortality in patients with TBI. Previous clinical trials have verified the beneficial effect of endogenous infusion of uric acid on preventing early poor progression and alleviating oxidative stress in ischemic stroke patients [35–37]. However, the SUA level after infusion of uric acid for neuroprotection was not investigated, and the correlation between SUA level and the occurrence of AKI was not evaluated in these trials. Consequently, it is worthwhile to evaluate whether the neuroprotective effect of uric acid supplement on the brain is greater than the adverse effect of developing AKI and discover a rational dose to avoid renal dysfunction in future study. This contradiction perfectly illustrates the principle that treatment aimed at brain injury should focus not only on the brain but also on other organs including the kidney.

## 5. Limitations

Firstly, because this was a single-center observational study, selection bias was unavoidable. Secondly, we did not collect the history of underlying diseases and record of using nephrotoxic drugs which may influence renal function and uric acid metabolism. Thirdly, AKI was not staged so that we could not discover the relationship between the SUA level and AKI stage. Finally, the time of the occurrence of AKI was not recorded so that we could not exactly evaluate the casual relationship between AKI and usage of drugs for reducing ICP.

## 6. Conclusions

SUA is an effective marker in predicting AKI following TBI. Combining SUA and serum creatinine is beneficial for physicians to evaluate renal function in TBI patients.

## Figures and Tables

**Table 1:** Baseline characteristics of AKI patients and non-AKI patients.

	Total (*n* = 374)	Non-AKI (*n* = 295)	AKI (*n* = 79)	*p*
Age (year)	43 (25-57)	41 (23-54)	47 (32-60)	0.010
Female (*n*, %)	93 (24.87)	76 (25.76%)	17 (21.52%)	0.433
Prehospital time (hour)	1 (1-2)	1 (1-2)	1 (1-1)	0.028
Vital signs in admission				
SBP (mmHg)	120 (105-138)	120 (106-138)	113 (102-133)	0.135
DBP (mmHg)	71.51 ± 17.103	72.34 ± 16.293	68.41 ± 19.641	0.104
Heart rate (bpm)	100 (81-119.25)	98 (80-117)	107 (89-124)	0.015
Temperature (°C)	36.7 (36.5-37.1)	36.7 (36.5-37)	36.8 (36.4-37.5)	0.368
Respiratory rate	20 (16-22)	20 (16-23)	18 (15-22)	0.022
GCS in admission	6 (5-9)	7 (5-10)	5 (4-6)	<0.001
Shock (%)	116 (31.02%)	71 (24.07%)	45 (56.96%)	<0.001
Laboratory tests				
WBC (10^9^/L)	14.34 (10.33-19)	13.98 (10.35-18.92)	14.98 (10.15-20.17)	0.607
Platelet (10^9^/L)	106.5 (68-171.25)	122 (76-180)	72 (44-111)	<0.001
Hemoglobin (g/L)	90 (76-110.25)	94 (79-112)	80 (71-96)	<0.001
Albumin (g/L)	31.60 ± 8.008	32.60 ± 7.867	27.86 ± 7.445	<0.001
Glucose (mmol/L)	9.49 (7.04-13.14)	8.84 (6.68-12.53)	11.7 (8.9-16.77)	<0.001
Chlorine (mmol/L)	110.9 (105.58-118.93)	109.3 (104.3-116.6)	116.9 (111.8-128.9)	<0.001
Cholesterol (mmol/L)	2.75 (1.97-3.62)	2.89 (2.22-3.68)	1.97 (1.35-2.79)	<0.001
Total bilirubin (*μ*mol/L)	14.6 (10.1-20.35)	13.9 (9.4-19.65)	17.5 (12.1-22.7)	0.001
Serum urea (mmol/L)	6.36 (4.93-8.64)	5.93 (4.65-7.77)	9.4 (7.11-15.3)	<0.001
Serum creatinine (*μ*mol/L)	71 (51-96.25)	65 (48-82)	133 (97-237)	<0.001
Uric acid (*μ*mol/L)	288 (186.75-388.5)	249 (165-347)	452.5 (360-585)	<0.001
Drugs for reducing ICP				
Hypertonic saline (%)	91 (24.33%)	81 (27.46%)	10(12.66%)	0.004
Mannitol (%)	257 (68.72%)	203 (68.81%)	54 (68.35%)	0.938
Glycerol fructose (%)	30 (8.02%)	23 (7.80%)	7 (8.86%)	0.760
Furosemide (%)	55 (14.71%)	39 (13.22%)	16 (20.25%)	0.129
In-hospital mortality (%)	187 (50%)	119 (40.34%)	68 (80.08%)	<0.001
90-day GOS	2 (1-3)	3 (1-4)	1 (1-1)	<0.001
Length of ICU stay (day)	2 (1-16)	2 (0-17)	2 (1-11)	0.384
Length of hospital stay (day)	11 (4-27)	13 (5-28)	6 (3-14)	0.001

**Table 2:** Multivariate logistic regression analysis of factors associated with AKI.

	OR	95% CI	*p*
Age	1.015	0.989-1.041	0.261
Prehospital time	0.730	0.482-1.104	0.136
Heart rate	0.984	0.969-0.999	0.041
Respiratory rate	0.982	0.911-1.060	0.646
GCS in admission	1.050	0.935-1.179	0.412
Shock	2.905	1.201-7.028	0.018
Platelet	1.000	0.991-1.009	0.963
Hemoglobin	0.995	0.975-1.015	0.612
Albumin	1.005	0.935-1.081	0.882
Glucose	1.078	0.993-1.170	0.073
Chlorine	1.018	0.982-1.055	0.341
Cholesterol	0.827	0.502-1.364	0.457
Total bilirubin	1.031	0.993-1.071	0.115
Serum urea	1.148	0.995-1.323	0.058
Serum creatinine	1.033	1.017-1.048	<0.001
Uric acid	1.008	1.004-1.011	<0.001
Hypertonic saline	0.789	0.247-2.525	0.690

OR: odds ratio; CI: confidence interval; GCS: Glasgow Coma Scale.

**Table 3:** Bivariate correlation analyses of uric acid and various clinical and laboratory parameters.

Variables	*r*	*p*
Age	0.088	0.090
Male	0.029	0.573
Prehospital time	-0.008	0.882
SBP	-0.051	0.325
DBP	-0.027	0.607
Heart rate	0.163	0.002
Temperature	-0.002	0.970
Respiratory rate	0.062	0.232
GCS in admission	-0.204	<0.001
Shock	0.204	<0.001
WBC	0.237	<0.001
Platelet	-0.264	<0.001
Hemoglobin	-0.030	0.562
Albumin	-0.126	0.015
Glucose	0.285	<0.001
Chlorine	0.286	<0.001
Cholesterol	-0.184	<0.001
Total bilirubin	0.037	0.478
Serum urea	0.291	<0.001
Serum creatinine	0.523	<0.001
Hypertonic saline	-0.158	0.002
Mannitol	-0.010	0.846
Glycerol fructose	-0.037	0.480
Furosemide	0.002	0.965

SBP: systolic blood pressure; DBP: diastolic blood pressure; GCS: Glasgow Coma Scale; WBC: white blood cell.

**Table 4:** Comparisons of the AUC value of different factors and models to predict AKI in the training cohort and validation cohort.

	AUC	Sensitivity	Specificity	Standard deviation	95% CI
Training set					
Uric acid	0.850	0.899	0.681	0.023	0.805-0.895
Serum creatinine	0.881	0.734	0.919	0.025	0.833-0.929
Uric acid+creatinine	0.917	0.823	0.871	0.019	0.880-0.954
Model 1	0.935	0.899	0.833	0.016	0.903-0.967
GCS	0.686	0.376	0.924	0.030	0.627-0.745
Validation set					
Uric acid	0.869	0.909	0.771	0.035	0.801-0.938
Serum creatinine	0.868	0.773	0.880	0.042	0.785-0.951
Uric acid+creatinine	0.905	0.773	0.904	0.035	0.836-0.974
Model 1	0.900	0.773	0.880	0.036	0.829-0.971
GCS	0.610	0.783	0.476	0.069	0.475-0.745

GCS: Glasgow Coma Scale; AUC: area under the ROC curve; CI: confidence interval. Model 1 is composed of heart rate, shock, serum creatinine, and uric acid.

## Data Availability

The data used and analyzed in this study are available from the corresponding author on reasonable request.

## Supplementary Materials

Supplementary materials The baseline characteristics of the training cohort and validation cohort.
