# Supplementary material for: The Predictive Value of Serum Uric Acid on Acute Kidney Injury following Traumatic Brain Injury
Source: Biomed Res Int. 2020 Aug 31;2020:2874369. doi: 10.1155/2020/2874369 (PMC7479452; doi:10.1155/2020/2874369)
Supplement: Supplementary materials — The baseline characteristics of the training cohort and validation cohort. [file 2874369.f1.docx]

| **Supplementary.** The baseline characteristics of training cohort and validation cohort | | | |
| --- | --- | --- | --- |
|  | training cohort (n=374) | validation cohort (n=105) | p |
| Age (year) | 43 (25-57) | 29 (44-54) | 0.433 |
| Female (n, %) | 93 (24.9%) | 25 (33.3%) | 0.824 |
| Prehospital time (hour) | 1 (1-2) | 1 (1-1) | 0.118 |
| Vital signs in admission |  |  |  |
| SBP (mmHg) | 120 (105-138) | 123 (111-140) | 0.119 |
| DBP (mmHg) | 72 (61-82) | 78 (68-88) | 0.004 |
| Heart rate (bpm) | 100 (81-119) | 100 (80.5-121) | 0.993 |
| Temperature (℃) | 36.7 (36.5-37.1) | 36.7 (36-37) | 0.010 |
| Respiratory rate | 20 (16-22) | 20 (15-22) | 0.169 |
| GCS in admission | 6 (5-9) | 4 (3-6) | <0.001 |
| Shock (%) | 116 (31.0%) | 28 (23.3%) | 0.369 |
| Laboratory tests |  |  |  |
| WBC (10^^^9/L) | 14.34 (10.33-19) | 15.03 (10.82-18.36) | 0.824 |
| Platelet (10^^^9/L) | 106.5 (68-171.3) | 90 (63.5-139.5) | 0.031 |
| Hemoglobin (g/L) | 90 (76-110) | 90 (79-105) | 0.875 |
| Albumin (g/L) | 31.9 (26.4-37.4) | 31.5 (28.4-35.6) | 0.636 |
| Glucose (mmol/L) | 9.49 (7.04-13.14) | 9.4 (7.25-13.18) | 0.969 |
| Chlorine (mmol/L) | 110.9 (105.6-118.9) | 114.9 (108.8-121.8) | 0.007 |
| Cholesterol (mmol/L) | 2.75 (1.97-3.62) | 2.57 (1.89-3.67) | 0.884 |
| Total bilirubin (umol/L) | 14.6 (10.1-20.4) | 13.3 (9.5-21.7) | 0.996 |
| Serum urea (mmol/L) | 6.36 (4.93-8.64) | 5.70 (4.19-7.80) | 0.013 |
| Serum creatinine (umol/L) | 71 (51-96) | 72 (58-116) | 0.163 |
| Uric acid (umol/L) | 288 (187-389) | 279 (166-399) | 0.501 |
| Drugs for reducing ICP |  |  |  |
| Hypertonic saline (%) | 91 (24.3%) | 26 (21.7%) | 0.928 |
| Mannitol (%) | 257 (68.7%) | 60 (50%) | 0.033 |
| Glycerol fructose (%) | 30 (8.0%) | 10 (8.3%) | 0.628 |
| Furosemide (%) | 55 (14.71%) | 20 (16.7%) | 0.289 |
| AKI (%) | 79 (21.12%) | 22 (18.3%) | 0.970 |
| In-hospital mortality (%) | 187 (50%) | 56 (46.7%) | 0.562 |
| 90-day GOS | 2 (1-3) | 1 (1-3) | 0.021 |
| Length of ICU stay (day) | 2 (1-16) | 5 (2-17) | 0.008 |
| Length of hospital stay (day) | 11 (4-27) | 9 (2-25) | 0.031 |
